# Editorial: Healthy grains and cereals: sustainability, new ingredients and innovative processing

**DOI:** 10.3389/fnut.2024.1412851

**Published:** 2024-05-02

**Authors:** Eveline Lopes Almeida, Elessandra da Rosa Zavareze, Marcio Schmiele

**Affiliations:** ^1^Planta Piloto de Cereais, Raízes e Tubérculos, Escola de Química, Centro de Tecnologia, Universidade Federal do Rio de Janeiro, Rio de Janeiro, Brazil; ^2^Laboratório de Biopolímeros e Nanotecnologia em Alimentos, Departamento de Ciência e Tecnologia Agroindustrial, Universidade Federal de Pelotas, Pelotas, Brazil; ^3^Laboratório Integrado de Cereais e Lipídios, Instituto de Ciência e Tecnologia, Universidade Federal dos Vales do Jequitinhonha e Mucuri, Diamantina, Brazil

**Keywords:** cold plasma, fermentation, fortification, germination, gluten, bioactive compounds, innovative processes, biosynthesis

The consumer of the current century, having more access to information and more accurate diagnoses, has guided the new directions of food processing. The quest for healthy foods that promote wellbeing and cater to specific dietary requirements has become the paramount objective in modern consumption patterns. Historically integral to human diets due to their nutrient richness and sensory appeal, grains have garnered renewed attention in contemporary nutrition, particularly with the revelation of their bioactive compound content. The traditional grain processing methods can be improved to meet new demands, which boosts scientific inquiry. This prompts researchers to explore innovative processing techniques and novel ingredients to enhance healthiness related to grain-based products. Articles within this Research Topic domain have delved into the following: How can advancements in processing technology and the integration of new ingredients can contribute to the development of healthier traditional grain-based foods?

Fermentation and germination, despite being old techniques used in food processing, can gain new meaning and be considered innovative processes for obtaining foods that promote health. Cho et al. studied the effectiveness of fermented grains (soybean, barley, job's tear, corn, wheat, and brown rice) using *Bacillus coagulans* in reducing visceral fat in obese individuals. The authors concluded that these fermented grains significantly reduced visceral fat in obese individuals, as well as decreased total body fat, body weight, and waist circumference, without the need for dietary or physical activity changes. Dong et al. provided an overview of the utilization of the Tartary buckwheat germination process, with an emphasis on biosynthesis mechanisms and enrichment in flavonoids. In addition to flavonoids, other phenolic compounds of significant interest can be enhanced by germination, such as phenolic acids (hydroxybenzoic and hydroxycinnamic) and non-flavonoids. The authors also show that emerging technologies such as ultra-high pressure, pulsed electric fields, cold plasma, microwaves, and ultrasound can be employed together with germination to improve the enhancement of these bioactive compounds.

The fortification technique of grain can provide new ingredients to supply deficient elements. Micronutrient malnutrition, which impacts health and productive life, affects a large number of people on the globe (1). In conjunction with the application of cold plasma, Starič et al. induced the biofortification of buckwheat sprouts with zinc during the maceration phase (hydropriming). They assessed it using micro-particle-induced-X-ray emission and X-ray fluorescence spectroscopy. The authors observed a better effect on zinc fortification without the use of cold plasma. Fortification of cereal flours with minerals can also occur through the blending of different flours. In this regard, Tura et al. used iron-rich dabi teff flour with germinated maize, roasted barley, roasted field pea, dehulled oats, and linseed to increase iron density in flour blends. The authors evaluated the iron bioavailability and concluded that the dabi teff and pea mixture yielded the best results, presenting itself as the best strategy with potential for combating childhood anemia in less developed countries.

New ingredients for food production that meet the needs of individuals who have metabolic disorders in relation to wheat are highly demanded. The research conducted by Guzmán-López et al. investigated the viability of utilizing six wheat lines, which had been genetically modified using RNA interference (RNAi) to exhibit reduced prolamin content, as prospective options for individuals afflicted with Wheat-dependent Exercise-induced Anaphylaxis (WDEIA). This study observed a decrease in gliadin content in all wheat lines modified through RNAi, concomitant with an increase in high-molecular-weight glutenin subunits (HMW-GSs) content compared to wild-type wheat. This reduction in gliadin content correlated with a decrease in IgE reactivity observed in the sera of patients diagnosed with WDEIA. However, there were differences in immune response patterns among patients and wheat genotypes.

Given so many possibilities for grain processing, this Research Topic focuses on current alternatives that can be used to change the chemical composition of grains and related products, enhancing the health benefits of their consumption. We hope that readers enjoy the topics covered and are motivated by the results of these researches.

## Author contributions

EA: Conceptualization, Supervision, Writing – original draft, Writing – review & editing. EZ: Validation, Visualization, Writing – original draft. MS: Validation, Visualization, Writing – original draft.
